# Trace: a high-throughput tomographic reconstruction engine for large-scale datasets

**DOI:** 10.1186/s40679-017-0040-7

**Published:** 2017-01-28

**Authors:** Tekin Bicer, Doğa Gürsoy, Vincent De Andrade, Rajkumar Kettimuthu, William Scullin, Francesco De Carlo, Ian T. Foster

**Affiliations:** 1 Mathematics and Computer Science Division, Argonne National Laboratory, 9700 South Cass Ave., Lemont, IL 60439 USA; 2 X-Ray Science Division, Advanced Photon Source, Argonne National Laboratory, 9700 South Cass Ave., Lemont, IL 60439 USA; 3 Computation Institute, University of Chicago and Argonne National Laboratory, 5735 South Ellis Ave., Chicago, IL 60637 USA; 4 Department of Computer Science, University of Chicago, 5801 South Ellis Ave., Chicago, IL 60637 USA; 5 Argonne Leadership Computing Facility, Argonne National Laboratory, 9700 South Cass Ave., Lemont, IL 60439 USA

**Keywords:** Tomography, Reconstruction, High-throughput, Big data

## Abstract

**Background:**

Modern synchrotron light sources and detectors produce data at such scale and complexity that large-scale computation is required to unleash their full power. One of the widely used imaging techniques that generates data at tens of gigabytes per second is computed tomography (CT). Although CT experiments result in rapid data generation, the analysis and reconstruction of the collected data may require hours or even days of computation time with a medium-sized workstation, which hinders the scientific progress that relies on the results of analysis.

**Methods:**

We present Trace, a data-intensive computing engine that we have developed to enable high-performance implementation of iterative tomographic reconstruction algorithms for parallel computers. Trace provides fine-grained reconstruction of tomography datasets using both (thread-level) shared memory and (process-level) distributed memory parallelization. Trace utilizes a special data structure called replicated reconstruction object to maximize application performance. We also present the optimizations that we apply to the replicated reconstruction objects and evaluate them using tomography datasets collected at the Advanced Photon Source.

**Results:**

Our experimental evaluations show that our optimizations and parallelization techniques can provide 158× speedup using 32 compute nodes (384 cores) over a single-core configuration and decrease the end-to-end processing time of a large sinogram (with 4501 × 1 × 22,400 dimensions) from 12.5 h to <5 min per iteration.

**Conclusion:**

The proposed tomographic reconstruction engine can efficiently process large-scale tomographic data using many compute nodes and minimize reconstruction times.

## Background

Synchrotron light sources enable the visualization of complex materials at very small scales, close to their molecular level ($\upmu \mathrm{m}$–nm). The current sensors and detectors at light sources can perform rapid data acquisition during the experiments at rates of thousands of frames per second (fps) with very high resolutions. For instance, the 2-BM (microCT) beamline at the Advanced Photon Source (APS) at Argonne National Laboratory (ANL) can collect 2000 fps with 2K $\times$ 2K pixels per frame, which translates to 16 gigabytes (GB) per second data generation rate with 16-bit pixels. These data generation rates are expected to increase by several orders of magnitude with upcoming upgrades in synchrotron light sources [1]. Even now, for large specimens, it is feasible to align and stitch together multiple frames to generate panoramas, which can increase the number of pixels in a 2D projection from 2K × 2K to 20K × 20K, increasing dataset size by 100 times.

Computed tomography (CT) is a common imaging method for collecting x-ray projections at synchrotron light sources. During CT experiments, multiple 2D projections are taken from different orientations of the target specimen, and then these projections are processed computationally to generate a 3D structure. The computational requirements of this tomographic reconstruction task vary according to both dataset size and the type of reconstruction algorithm used. Two common reconstruction methods are *analytical reconstruction*, including *filtered back-projection* (FBP), and *iterative reconstruction*. FBP methods, such as Gridrec [2], perform only a single pass over the input projection dataset and therefore require significantly less computation than do iterative reconstruction algorithms, which may need tens or even hundreds of iterations. However, several critical issues arise with the application of FBP methods that affect the quality of reconstructed images. First, FBP requires many projections; if the number of projections is insufficient, then FBP can introduce artifacts in the reconstructed image. Second, since FBP requires a higher number of projections, the target specimen is exposed to a greater radiation dose, which may be infeasible if the specimen (e.g., a biological sample) is dose-sensitive. Third, analytical reconstruction techniques are susceptible to errors and noise in data, which are common due to the experimental limitations.

**Fig. 1:**
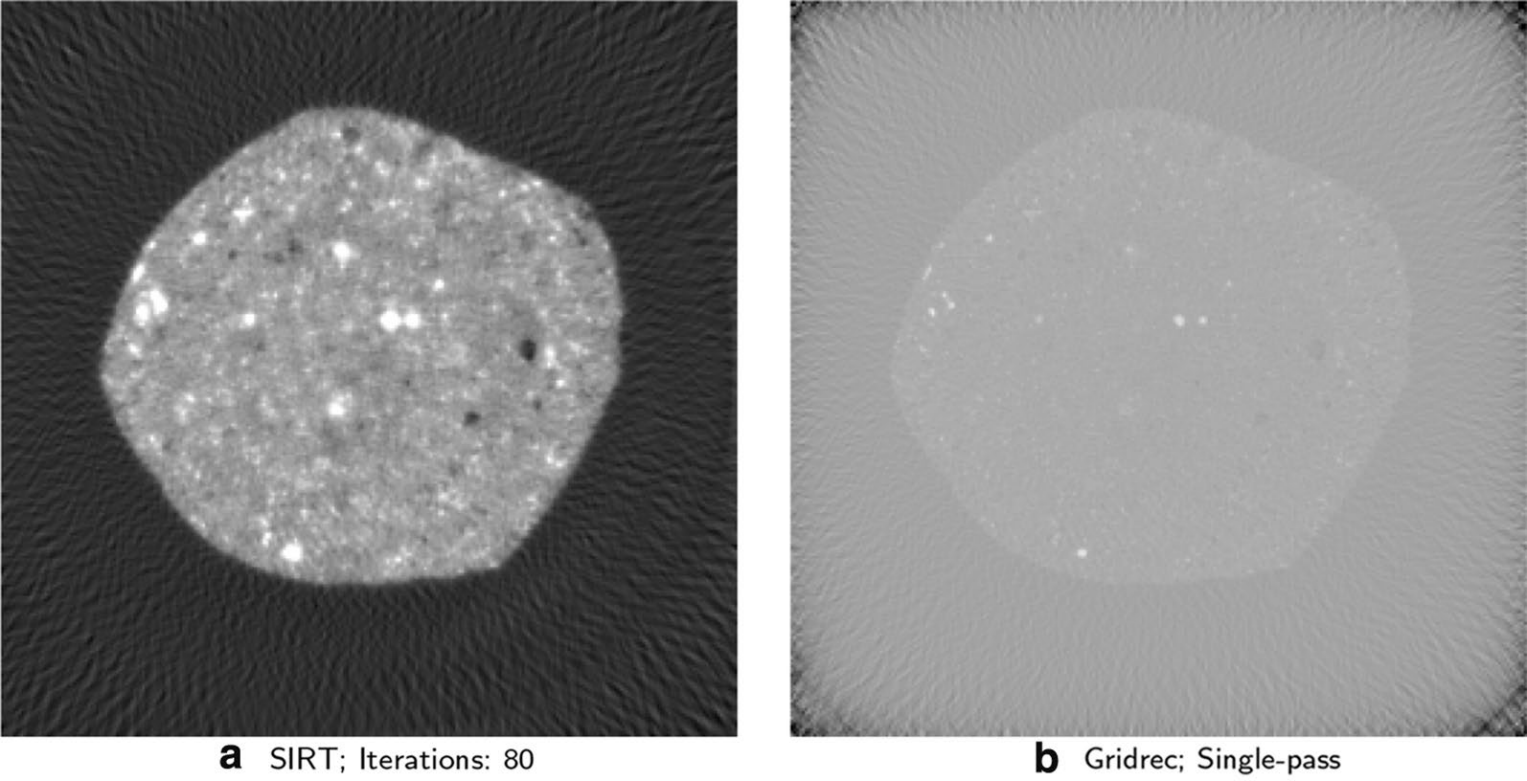
Reconstructed 3D image of a shale sample [46]. The input dataset consists of 90 projections each with 2K × 2K pixels. **a**, **b** The 3D reconstructed image using SIRT and Gridrec algorithms, respectively. Reconstruction with SIRT takes $\sim$353 s for 80 iterations, using two threads, reconstruction with Gridrec takes only $\sim$9 s, using one thread. However, SIRT with 80 iterations provides a higher-quality image than does Gridrec

In contrast, the iterative reconstruction algorithms on which we focus in this paper can provide better 3D images, albeit at the cost of additional computing power (see Fig. 1). Specifically, iterative algorithms such as Arithmetic Reconstruction Technique (ART), Maximum Likelihood Expectation Maximization (MLEM), Simultaneous Iterative Reconstruction Technique (SIRT), and Penalized Maximum Likelihood (PML) [3] use statistical models and cost functions to iteratively converge to a refined solution consistent with the measured data. Further, these methods can operate effectively with fewer projections, resulting in less dose exposure to specimens [4, 5].

In this paper, we focus on parallelization methods for efficient iterative tomographic reconstruction. We describe methods that make it possible to provide timely feedback to experimentalists (within minutes, and indeed with enough processing power, seconds), even for extremely large datasets. This work builds on and extends our previous research [6], with the following new contributions. First, we enable the reconstruction of *a* sinogram by *multiple nodes*, using distributed-memory parallelization. Distributed memory parallelization, in addition to shared memory parallelization from our previous work, lets users reconstruct very large datasets in a timely manner. Second, we analyze the effect of data organization and structures, and perform cache-sensitive execution of reconstruction tasks. Third, we extensively evaluate our optimizations and present the cost of different phases during execution.

The remainder of this paper is organized as follows. We discuss the related works in “Related work” section. Then, we introduce our middleware, Trace, and its optimizations in “High-performance iterative tomographic reconstruction” section. We evaluate and present the performance of Trace with medium- and large-scale datasets in “System evaluation” section, and conclude in “Conclusion” section.

## Related work

The parallelization of iterative reconstruction algorithms has been researched in different areas [7–11]. Although these works show satisfactory reconstruction performance, most of them focus on improving the performance of a specific reconstruction algorithm with shared memory parallelization. In our work, we consider easing the implementation and parallelization of different reconstruction algorithms using a MapReduce-like middleware [6, 12, 13], and scale reconstruction operations to many compute nodes.

Manycore architectures, such as GPUs, have been extensively used for iterative reconstruction [14–17]. Especially in medical imaging, iterative reconstruction approaches are used for generating high-quality 3D images [18–20]. Although GPUs can provide high computational throughput, the analysis code is typically tailored for a specific device and application. Moreover, GPUs can accommodate only small datasets and are not suitable for large-scale tomography data. Trace enables efficient reconstruction of large-scale datasets on multicore clusters where adequate memory is available.

Domain decomposition techniques have been used for parallelization of reconstruction operations [21, 22]. We perform decomposition at the sinogram space, while considering the distribution and synchronization of reconstruction tasks on many physical nodes. In a recent work, Wang et.al. highlight the long execution times of iterative reconstruction approaches and address the cache utilization issues of model-based iterative reconstruction (MBIR) [23, 24] using optimized buffers called supervoxel. Their approach mainly addresses the cache utilization issues; however, the scalability of reconstruction tasks on large number of compute nodes is not considered.

Data analysis and workflow management at synchrotron light sources have gained a lot of importance in recent years [25–28]. CAMERA, for instance, is an interdisciplinary project at Lawrence Berkeley National Laboratory [29], which investigates problems of DOE user facilities and develops fundamental new mathematical solutions. Another similar effort is also initiated at Brookhaven National Laboratory to ease the data analysis tasks for NSLS-II facility users [30]. Most of these projects aim to provide timely data analysis for beamline users [31, 32]. Our data analysis tasks and workflows rely on a MapReduce-like processing structure for efficient and scalable processing. Since MapReduce lets users easily customize Map and Reduce phases, the integration of other reconstruction and analysis algorithms, such as Discrete Algebraic Reconstruction Technique [33], Total Variation [34], and Sparse Reconstruction[35] between (and during) iterations, is possible.

Although other MapReduce implementations, such as Spark [36] and Hadoop [37], can provide scalability and fault tolerance, they are tailored to commodity hardware and cannot perform efficient execution on high-performance computing resources. Our middleware utilizes the replicated reconstruction objects which enables reconstruction tasks to scale tens of thousands cores on high-performance computing resources and provide timely turnaround times for compute-intensive works [6, 38]. We provide the details of our middleware in the following section.

## High-performance iterative tomographic reconstruction

In this section, we first provide some background on the organization of tomographic datasets and iterative reconstruction techniques, and then, we present the components and execution flow of our middleware.

### Tomographic data acquisition and organization

During tomographic data acquisition, a detector collects 2D projections from different rotations ($\theta$s). This process generates a 3D dataset with (*z*,* y*,* x*) dimensions, where* z*,* y*, and* x* represent projections (angular dimension), sinograms, and columns (spatial dimensions), respectively. Each value (pixel) in the dataset, which is generally a 16-bit unsigned integer, is a line integral of an X-ray passing through the target object from a specific angle $\theta$.

Since each 2D projection represents the same object from a different $\theta$, projections as a collection can be used to reconstruct a 3D image of the target object. Typically, the dimensions of a reconstructed 3D image follow a (*y*,* x*,* x*) pattern. For example, a tomography dataset with dimensions (720, 512, 2048) yields a 3D image with dimensions (512, 2048, 2048). Note that each sinogram corresponds to a slice in 3D image; that is, a one-to-one relationship exists between sinograms and slices. This relationship is sufficient for performing parallel processing on y dimension for unregularized reconstruction algorithms. In this paper, we focus on iterative tomographic reconstruction algorithms, where unknown coefficients in a 3D image are converged to a refined solution at each iteration.

Iterative reconstruction algorithms consist of two main computational stages: *forward* and *back projection*. During the forward projection, a *simulated data* value is computed for each ray. The computation of simulated data depends on previous iteration’s voxel values and ray-lengths on intersected voxels. For instance, SIRT algorithm computes $d_r=\sum _{(i) \in V} m_{i}\times l_{i}$ while calculating the simulated data value ($d_r$) of a ray (*r*). Here, $m_{i}$ is the value of voxel with index *i* on reconstructed image; $l_{i}$ is the length of *r* on voxel$_{i}$; and *V* is a set of voxel indices that are visited by *r*. Since the number of simulated rays and voxels can be very large, forward projection requires large-scale compute resources. After forward projection computation, back projection is performed. During this stage, the simulated data values of *all* the rays that pass through the voxel$_{i}$ are used for computing weight values, $w_{i}$. Later, $w_{i}$ values are normalized with $l_{i}$, and update operations on 3D image voxels are performed. Parallelization at the sinogram level is typically straightforward, since all the rays can sequentially be simulated on a sinogram and each sinogram can be reconstructed independently. However, in-sinogram (or in-slice) parallelization, where a single sinogram is processed by many processing units, is nontrivial. This is mainly due to the data dependencies between rays’ simulated data and visited voxels ($d_r$ computation), and $w_{i}$ computation.

### Parallelization of iterative reconstruction using distributed and shared memory techniques

**Fig. 2:**
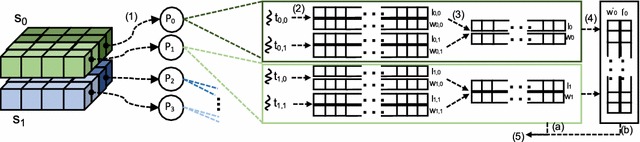
Execution flow (steps 1–5) of Trace middleware with sinogram-level group communication

Figure 2 presents our approach to parallelize iterative reconstruction algorithms in Trace. Trace performs iterative reconstruction in several steps. First, the ray-sum values that intersect the same plane, that is, sinogram (*y* dimension), are equally distributed among processes, $P_j$ (step 1). For instance, in Fig. 2, the tomographic dataset consists of two sinograms, $s_0$ and $s_1$, and these sinograms are evenly distributed between $P_{0...3}$.

Each $P_j$, initiates a number of threads, $t_{j,k}$ that then compute the $w_{i}$ and $l_{i}$ values. In Trace, these values are derived by using a modified version of Siddon’s algorithm [39]. There are many iterative reconstruction algorithms that perform different computations in forward and backprojection stages. These computations typically result in different $l_i$ and $w_i$ values. Trace provides an API that makes it easy for developers to implement their algorithms for forward and backprojection kernels. Specifically, users can develop and parallelize customized reconstruction algorithms by extending the Reduce(...) and Update(...) functions in API, which correspond to parallel forward and backprojection kernels, respectively. During step 2, Trace runtime system automatically applies the user-selected (or user-implemented) Reduce(...) function to the $l_i$ and $w_i$ arrays. Trace uses a wrapper data structure called *replicated reconstruction object* (replica) for the management of $l_i$ and $w_i$. It is important to note that the parallelization techniques in Trace rely on full replication, that is, each thread works on its own replica [40]. This parallelization technique also lets Trace scale up to the number of ray-sum values in input dataset; therefore, it provides fine-grained reconstruction. On the other hand, since each thread requires a private replica, memory utilization can be high which may limit the level of parallelization.

After all rays are processed and new length and weight values in replicas are computed, threads perform *local combination* (step 3). During this phase, threads that operate on the same sinogram synchronize and combine their replicas. This phase leads to a single reconstruction object per process. If the number of sinograms, $n_s$, in the tomography dataset is larger than (or equal to) the number of initiated processes, $n_p$, then the Trace runtime system starts updating the corresponding 3D image slices (with Update(...) function) using locally combined replicas (shared memory parallelization) and proceeds to the next iteration (step 5.a).

Shared memory parallelization alone can provide sufficiently good performance for many tomography datasets [41–43]. However, it is still limited with parallel reconstruction of a sinogram on a single compute node, i.e., $n_s \ge n_p$. For very large datasets, such as that of the mouse brain [44], reconstruction of a single sinogram can take hours to finish. Therefore, a higher level of parallelization, where a compute node can perform reconstruction with part of a sinogram, is needed. This type of data parallelization requires a combination of shared and distributed memory parallelization, and thus both thread- and process-level synchronizations. Specifically, if there are more processes than sinograms, that is, if $n_p>n_s$, then processes that operate on *the same sinogram* perform interprocess synchronization to compute refined reconstruction object values. Trace automatically manages this group-level synchronization using sinogram identifiers (e.g., $s_0$ and $s_1$). Figure 2 illustrates this process with $P_0$ and $P_1$, which operate on the same sinogram, $s_0$. $P_0$ and $P_1$ can start the next iteration only after the *group combination* phase (step 4).
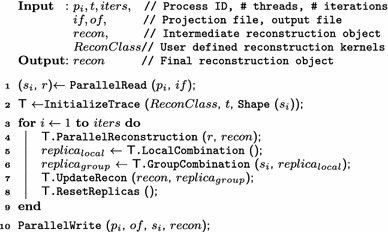


Algorithm 1 presents the pseudocode of this process. At line 1, the system reads corresponding sinograms according to process IDs ($P_j$). At line 2, the Trace middleware is initialized with ReconClass, number of threads (*t*) and sinogram shape (Shape($s_i$)). ReconClass wraps user defined Reduce(...) and Update(...) functions that will be applied to the input data. The Trace middleware allocates and initializes *t* number of replicas, and assigns each thread to a replica. Recall that replica sizes are determined according to dimensions of the assigned sinograms, hence dimension information of the sinogram is also passed to the middleware. Lines 2 and 3, perform shared memory parallelizations in which ParallelReconstruction(...) updates the replicas using user defined functions. LocalCombination(...) combines the replicas and generates a local intermediate replica. Then, this replica is further combined with replicas from other processes (distributed memory parallelization) using GroupCombination(...). Finally, the resulting replica is used for updating local *recon* object.

While this hybrid parallelization method significantly improves the scalability of reconstruction process, it can also introduce some overhead. In particular, if the replicated reconstruction objects are large, the communication overhead between processes becomes more visible (mainly because of the process-level group combination operations). This overhead is extensively evaluated with different tomography datasets in “System evaluation” section.

### Improving the cache utilization of Trace

**Fig. 3:**
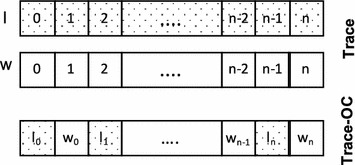
Data organization replicated reconstruction object: Trace and cache optimized Trace-OC

Another important issue for efficient reconstruction is the data access pattern, which affects cache utilization. We transform the independent $w_i$ and $l_i$ arrays in replicas from a *struct of arrays* (SoA) to an *array of structs* (AoS) to improve cache utilization. Figure 3 represents the organization of both data structures. The initial replica implementation in Fig. 3 treats both arrays independently, that is, SoA representation is used, whereas Trace-OC combines both arrays and performs data accesses on AoS. Specifically, since most of the data accesses rely on voxels, we reorganize *w* and *l* values with respect to their corresponding voxels; thus, accessing to one of the voxel variables results in loading both *w* and *l* values into the cache. This transformation improves both temporal and spatial data locality in Trace and provides better cache utilization. Note that, it is typically preferable to use SoA representation, where consecutive data access pattern to an array of elements is observed. However, since we have irregular data access pattern during reconstruction, AoS provides better cache utilization. In the next section, we analyze the impact of large replicas and cache optimizations on overall execution time.

## System evaluation

We evaluated our system on Cooley, a visualization cluster located at Argonne National Laboratory [45]. Cooley has 126 compute nodes, where each node consists of 12 cores (two 2.4 GHz Intel Haswell CPUs, each with 6 cores). Moreover, each node has 384 GB of memory for large-scale data visualization and analysis. The compute nodes are connected with FDR InfiniBand for high-performance communication.

Trace provides four different iterative reconstruction algorithms, ported from the TomoPy package [3]: MLEM, SIRT, PML, and Accelerated PML Reconstruction (APMLR). We present here results on the performance of our system using SIRT. Considering the dimension parameters that were introduced in “High-performance iterative tomographic reconstruction” section, the computational complexity of SIRT algorithm is $O(N_{z}\times N_{y}\times N_x^2)$ per iteration.

We used three tomography datasets to evaluate the performance of our middleware. Two are real experimental data collected at APS beamlines: a mouse brain dataset [44] and a shale sample [46]. The mouse brain dataset is a large tomography dataset that consists of 4501 projections, 22,400 sinograms, and 22,400 columns, which requires $\sim$4.2 TB disk space. Moreover, the reconstructed 3D image’s dimensions are 22,400 $\times$ 22,400 $\times$ 22,400, where each voxel is a single-precision floating-point number. The total size of the reconstructed mouse brain image is $\sim$40.9 TB. The shale sample is a medium-sized dataset and includes 1440 projections, 2048 sinograms, and 2048 columns. The total size of the shale data is $\sim$12 GB, and its corresponding reconstructed 3D image size is $\sim$32 GB. We also used simulation data to evaluate system performance with varying numbers of projections and column sizes.

### Cache-sensitive iterative reconstruction

In our first set of experiments, we evaluate the serial (non-parallelized) performance of our system on a single compute node. This version of our system uses only a *single core* during reconstruction.

**Fig. 4:**
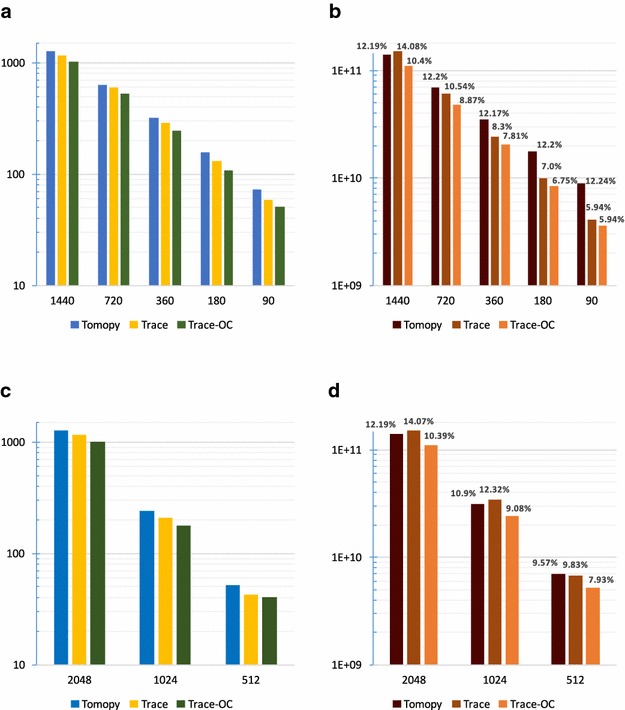
Execution times (secs) and L1 cache misses with respect to different numbers of projections and columns using SIRT. **a** Execution times with varying numbers of projections. **b** Number od L1 cache misses with varying number of projections. **c** Execution times with varying number of columns. **d** Number of L1 cache misses with varying number of columns

In Fig. 4a, we present the reconstruction times (*y*-axis, in log scale) of a single sinogram with respect to varying numbers of projections (*x*-axis). The total size of the columns is set to 2048. Thus, for instance, when the number of projections is *P* = 1440 (first set of bars in Fig. 4a), the dataset dimensions are 1440 $\times$ 1 $\times$ 2048. For all configurations, we set the number of iterations to five.

We performed the reconstructions using the original TomoPy and two different versions of our middleware: the version without any optimizations, Trace; and the optimized cache-sensitive version, Trace-OC. In all configurations, Trace-OC outperforms both TomoPy and Trace. Compared with TomoPy, the speedups with Trace-OC range from 1.19 to 1.44, which effectively reduce the execution times by up to 30%. Compared with Trace, the speedups with Trace-OC are from 1.12 to 1.21, which result in up to $\sim$18% reduction in reconstruction time.

The main reason for this performance increase is better cache utilization in Trace-OC. Specifically, since both the temporal and spatial localities of the *length* and *weight* values are improved with the alternative data organization, the reconstruction operations incur fewer number of cache misses. We present the L1 cache misses in Fig. 4b to show the correlation between reconstruction times and the cache misses. Since L1 is the first level cache that buffers the data for processing, the miss rate at L1 cache has significant effect on overall performance of application. Here, the *y*-axis shows the total number of L1 cache misses, whereas the percentages are the ratio between cache misses and the total number of requests to the cache (i.e., the sum of both hits and misses at L1 cache). In general, the smaller number of cache misses results in shorter execution times. Similarly, the lower ratios between L1 cache misses and hits (miss ratios) indicate better cache utilization. The only outlier configuration to this generalization is Tomopy with 1440 projections, where both the number of L1 cache misses and percentage values are smaller than the Trace configuration. In this case, last level cache (LLC) bandwidth of Trace is higher than that of TomoPy (58.9 vs 55.1 million loads per second), which favors Trace performance.

In Fig. 4c, d, we profile the reconstruction times and cache utilization, respectively, with varying column sizes (*x*-axis). We set the number of projections to 1440 for all configurations and reconstruct a single sinogram. The performance improvements follow the same trend as before. Specifically, Trace-OC provides speedups that range from 1.29 to 1.35, with up to $\sim$26% shorter execution times than with TomoPy. Similarly, compared with Trace, the observed reduction in execution times with Trace-OC is between 6.3 and 14.4%.

If we compare Fig. 4a, c, we see that the reconstruction times are more sensitive to column sizes than to the number of projections. Specifically, when the number of the projections is doubled, the execution times also double; in other words, we observe a linear increase. When the column sizes are doubled, however, the reconstruction times show an almost exponential increase. The main reason for this higher sensitivity to column sizes (i.e., *x* dimension) is the relationship between the number of variables in input dataset $O (N_z\times N_y \times N_x)$ and output 3D image $O (N_{y}\times N_x^2)$ [13].

### Parallel reconstruction of medium- and large-scale tomography data

We next compare the execution times taken in the different phases of the reconstruction for medium- vs large-sized datasets. As in the preceding section, we reconstructed a single sinogram from each dataset and set the total number of iterations to 5 and 40 for brain and shale datasets, respectively. We used 32 Cooley nodes (32 $\times$ 12 = 384 cores) for the computation. This type of reconstruction requires sharing and processing one sinogram with multiple nodes and therefore needs both inter- and intra-node synchronization among processes and threads. We break down the execution times to observe performance issues during reconstruction.

**Fig. 5:**
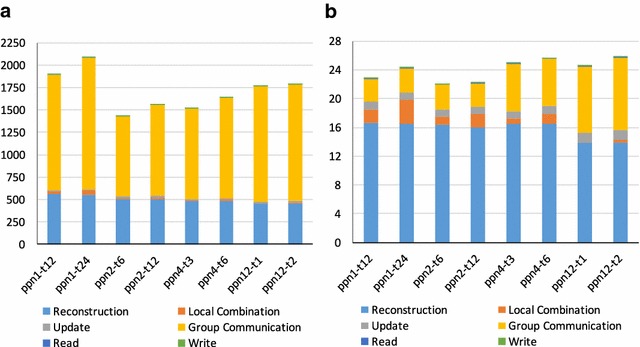
Breakdown of iterative reconstruction times (secs) with respect to varying parallelization configurations. Here ppn stands for *processes per node*, and t is the number of initialized *threads per process*. For example, with configuration ppn2-t6, Trace-OC initiates two processes on each compute node, each with six worker threads (i.e., a total of 12 threads per compute node). We used 32 compute nodes for the reconstruction. **a** Mouse brain, **b** shale sample

In Fig. 5a, we show execution times when processing a mouse brain sinogram. We use different numbers of processes and threads for each configuration in order to observe their effect on performance. We format the label of each configuration as ppn#-t#, in which ppn# refers to the number processes that are initiated in *each compute node* and t# is the number of threads in *each process*. For instance, the ppn2-t6 configuration initiates two processes in each node, where each process runs six threads. Therefore, the total number of active processes during the reconstruction is 32 $\times$ 2 = 64, and the total number of threads is 64 $\times$ 6 = 384.

For each configuration, we divide the execution times into six phases. In the Reconstruction and Update phases, forward and back projections are computed. In the Local Combination phase, threads in a process perform shared memory synchronization and exchange/reduce intermediate values, namely forward projection values, inside a node (intra-node synchronization). During the Group Communication phase, the processes that work on the same sinogram exchange the locally reduced values (inter-node synchronization). The Read and Write phases correspond respectively to the reading time of the sinogram dataset (input file) and writing time of the reconstructed 3D image (output file).

#### Performance analysis of parallel large-scale sinogram reconstruction

Our first observation from Fig. 5a is that the Group Communication dominates the overall execution time for all configurations. The main reason for this behavior is the large replicas that are exchanged during the inter-node communication. Specifically, the size of a replica is 2$\times$ larger than the size of a 3D image slice, since it accommodates $l_{i}$ and $w_{i}$ values. Recall that each 3D image slice requires an array with 1 $\times$ 22,400 $\times$ 22,400 dimensions for the mouse brain, which is $\sim$2 GB (single-precision floating-point numbers). Since a locally combined replica ($replica_{local}$) is 2$\times$ larger than 3D slice, its size is 4 GB. If we consider ppn4-t* configuration, where the total number of processes is 32 $\times$ 4 = 128 and number of iterations is 5, the total exchanged data are (at least) 5 $\times$ 128 $\times$ 4 = 2.5 TB throughout the execution. This data movement introduces significant overhead. Specifically, Group Communication corresponds to 63.8–72.3% of the total execution times in Fig. 5a, in which the minimum communication time occurs with the ppn2-t6 configuration. We suspect that this configuration provides good data and process locality for compute nodes, where each node consists of two CPUs and each CPU has six cores.

Looking next at the Reconstruction phase, we see that all configurations follow similar trends, with the ppn12-t1 configuration being the most efficient. Since this configuration maps each process to a core, it provides the most isolated environment for the processes and provides the highest throughput. Considering the overall execution time, however, we observe that ppn2-t6 is the most efficient configuration, since it provides above-average reconstruction time with better communication performance.

If we compare ppn2-t6 with other configurations, ppn2-t6 shows speedups ranging from 1.12 to 1.49. Note that, these speedups are all based on end-to-end processing time of a single sinogram using 32 compute nodes. The end-to-end execution time of the same dataset with a single core is more than $\sim$63 h, which means that ppn2-t6 can provide 158$\times$ speedup relative to the best single-core (sequential) performance. Since the mouse brain dataset consists of 22,400 sinograms, iterative reconstruction with a single core is not feasible, especially considering that many of iterations are needed for high-quality 3D images.

#### Performance analysis of parallel medium-scale sinogram reconstruction

Figure 5b shows results for the same experiments with a shale sample. Since the shale dataset is smaller than that of the mouse brain, Group Communication introduces much less overhead. Therefore, the Reconstruction phase becomes the dominating factor, which corresponds to 52–73% of the total execution time. As in the previous experiments, we observe the best total execution time with the ppn2-t6 configuration, even though ppn12-t1 shows the best Reconstruction time.

In the Local Combination phase, we observe that the configurations with more threads—ppn1-t12, ppn1-t24, and ppn2-t12—require more time than the other configurations. Since all the threads that belong to same process need to synchronize after updating their replicas, synchronization time increases with larger number of threads. The maximum thread synchronization overhead is 12.2%, which is observed in the ppn1-t24 configuration. We see a similar trend in Fig. 5a, though the Local Combination phase is mostly dominated by communication and reconstruction times.

#### Analysis of large-scale parallel reconstruction with strong scaling

**Fig. 6:**
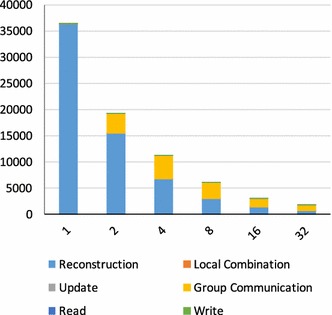
Execution times (s) of reconstructing a single sinogram mouse brain with different numbers of compute nodes. The number of iterations is set to five and the ppn2-t6 configuration is used

Figure 6 shows how execution times for the mouse brain dataset scale with different numbers of compute nodes when using the ppn2-t6 configuration. As in the previous experiments, the Reconstruction and Group Communication phases dominate overall execution times. Specifically, the reconstruction phases take the most time on up to 8 nodes, then (for 16 and 32 nodes) communication cost becomes dominant. The main reason for this change is that while the computation parallelizes almost perfectly, communication does not; and thus, while it reduces in absolute terms as we scale from 2 to 32 nodes, it increases as a percentage of total time, from 19.7 to 60.1%. Nevertheless, we still achieve a speedup of 21.6 on 32 nodes relative to 1 node.

## Conclusion

In this paper, we have presented our middleware, Trace, which provides a framework for high-performance implementation of iterative tomographic reconstruction algorithms. It enables the fine-grained parallelization of reconstruction algorithms using shared and distributed memory parallelization techniques, where a single sinogram can be reconstructed by many processes. Further, we optimize the cache utilization of reconstruction by transforming replicated reconstruction objects, in which we reorganize data structures according to application’s data access pattern.

We evaluated our methods using simulated and real-world tomography datasets, and presented execution times of different phases. Our experimental results showed that the proposed methods can provide up to 158 $\times$ speedup (using 32 compute nodes) over single-core configuration, which decreases the end-to-end processing time of a sinogram (with (4501, 1, 22,400) dimensions) from $\sim$12.5 h to <5 min per iteration.
